# Supplementary material for: Regnase-1-mediated post-transcriptional regulation is essential for hematopoietic stem and progenitor cell homeostasis
Source: Nat Commun. 2019 Mar 6;10:1072. doi: 10.1038/s41467-019-09028-w (PMC6403248; doi:10.1038/s41467-019-09028-w)
Supplement: Supplementary file 1 — Supplementary Information [file 41467_2019_9028_MOESM1_ESM.pdf]

Supplementary Information

Regnase-1-mediated post-transcriptional regulation is essential for hematopoietic stem and progenitor cell homeostasis

Kidoya et al.

Supplementary Figure 1

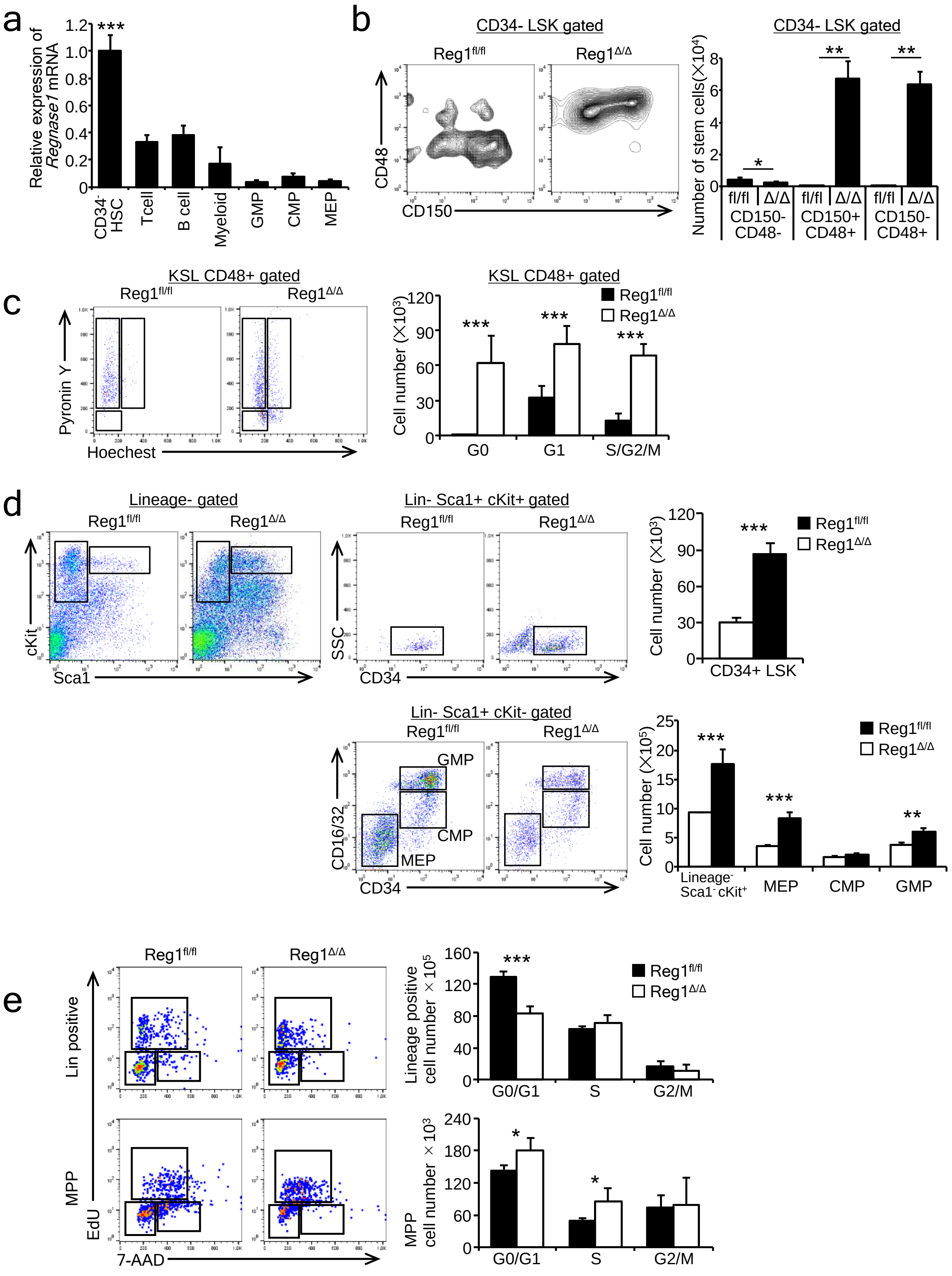

Supplementary Figure 1. Expression of Regnase-1 and the effect of Regnase-1 deficiency on the cell cycle in progenitors and differentiated blood cells.

(**a**) *Regnase-1* mRNA expression in LSK CD34$^-$ Flt3$^-$ (CD34$^-$ HSC), CD45$^+$ CD3$^+$ cells (T cells), CD45$^+$ B220$^+$ (B cells), CD45$^+$ CD11b$^+$ cells (Myeloid), Lin$^-$ cKit$^+$ Sca1$^-$ CD34$^+$ CD16/32^{high} (GMP), Lin$^-$ cKit$^+$ Sca1$^-$ CD34$^+$ CD16/32^{mid} (CMP) and Lin$^-$ cKit$^+$ Sca1$^-$ CD34$^-$ CD16/32^{low} (EMP) cells from BM of 8 week-old adult mice (n = 3 per group; 2 independent experiments). (b) Representative flow cytometric analysis of CD34$^-$ LSK cells ´ CD48 and CD150 expression in 8 week-old control (fl/fl) or Regnase-1-KO (Δ/Δ) mice (n=3 per group; 2 independent experiments). Quantification of CD150$^+$ CD48$^-$, CD150$^+$ CD48$^+$ and CD150$^-$ CD48$^+$ cells of control (fl/fl) and Regnase-1-KO (Δ/Δ) mice. (c) Representative cell cycle plots of CD48$^+$ LSK populations in BM from control (fl/fl) or Regnase-1-KO (Δ/Δ) mice using Hoechst 33342/pyronin Y staining (n =3 per group; 2 independent experiments). Graph showing number of cells in the G0, G1 and S/G2/M phases. (d) Flow cytometric analysis of CD34$^+$ LSK, Lin$^-$ cKit$^+$ Sca1$^-$ cells, Lin$^-$ cKit$^+$ Sca1$^-$ CD34$^+$ CD16/32high (GMP), Lin$^-$ cKit$^+$ Sca1$^-$ CD34$^+$ CD16/32mid (CMP) and Lin$^-$ cKit$^+$ Sca1$^-$ CD34$^-$ CD16/32low (EMP) cells in BM of mice 4 weeks after transplantation of control (fl/fl) or Regnase-1-KO (Δ/Δ) KSLs (n=3 per group; 2 independent experiments). Quantification of CD34$^+$ LSK, Lin$^-$ cKit$^+$ Sca1$^-$ cells, GMP, CMP and MEP in bone marrow MNCs. (e) Cell-cycle analysis of control (fl/fl) or Regnase-1-KO (Δ/Δ) BM Lin$^+$ cells and MPP populations by flow cytometry using EdU/7AAD staining. Dot plots indicating the frequency of HSC in G0/G1 (EdU$^-$ 7AAD$^-$), G2/M (EdU$^+$), or S (EdU$^+$ 7AAD$^+$) phase of the cell cycle (n =3 per group; 3 independent experiments). Error bars indicate mean ± SD. *p < 0.05; **p <0.01, ***p < 0.005, t-test.

Supplementary Figure 2

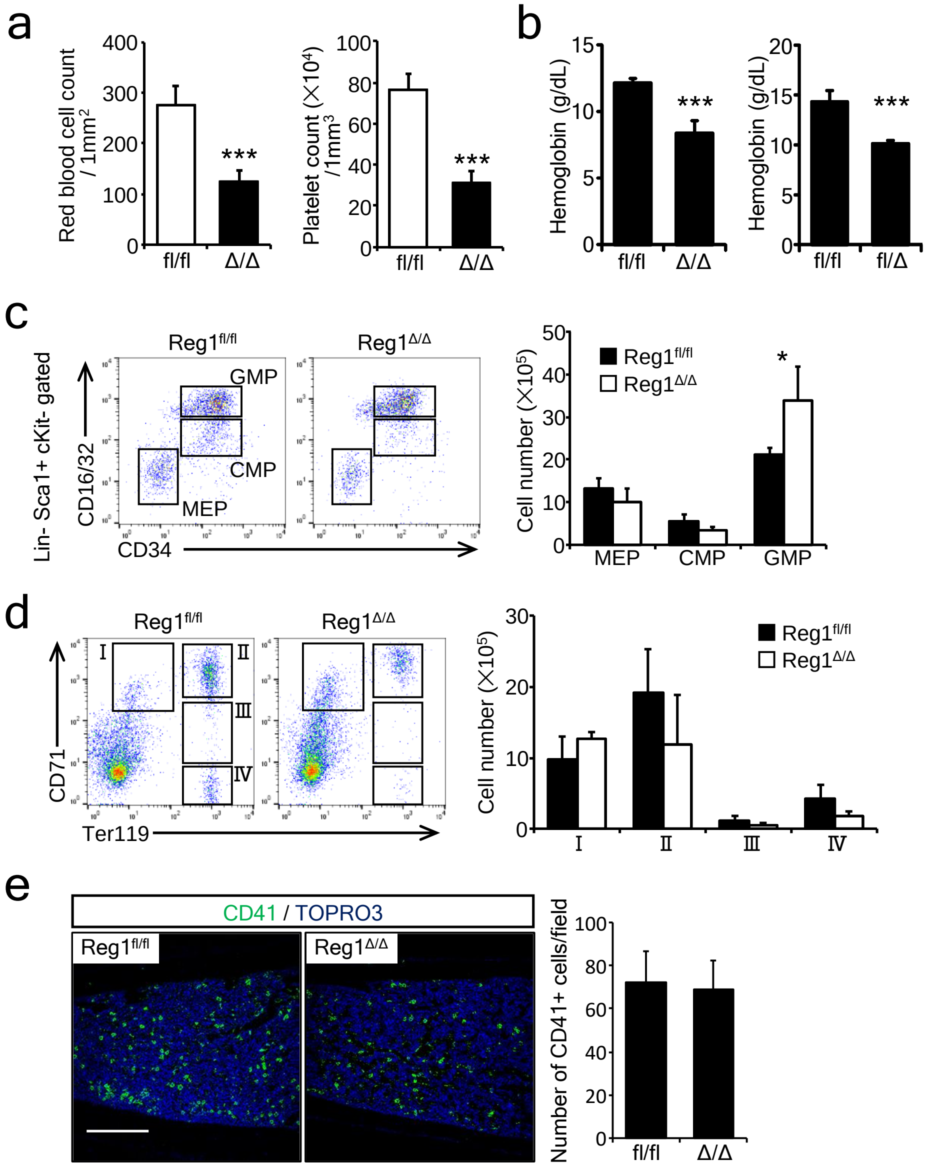

Supplementary Figure 2. Analysis of erythro-megakaryopoiesis in Regnase1-KO mice.
(**a**) Peripheral red blood cell and platelet counts in blood samples from 8 week-old control (fl/fl) or Regnase-1-KO (Δ/Δ) mice. (n =3 per group; 2 independent experiments). (**b**) Peripheral blood hemoglobin levels of 8 week-old control (fl/fl) and Regnase-1-KO (Δ/Δ) mice (right graph) or 12 month-old control (fl/fl) and Regnase-1 hetero mutant (flox/Δ) mice (left graph) (n =3 per group; 3 independent experiments). (**c**) Flow cytometric analysis of Lin^- $cKit^+$ $Sca1^-$ $CD34^+$ $CD16/32^{high}$ (GMP), Lin^- $cKit^+$ $Sca1^-$ $CD34^+$ $CD16/32^{mid}$ (CMP) and Lin^- $cKit^+$ $Sca1^-$ $CD34^-$ $CD16/32^{low}$ (EMP) cells in BM from 8 week-old mice as indicated (n=3 per group; 2 independent experiments). Quantification of GMP, CMP and MEP in bone marrow MNCs. (**d**) Flow cytometric analysis of erythroid progenitors in BM from 8 week-old mice as indicated (n =3 per group; 2 independent experiments). Quantification of erythroid subsets in bone marrow. (**e**) Immunohistochemical staining of $CD41^+$ megakaryocytes in control and Regnase-1-KO (Δ/Δ) BM (n =3 per group). The scale bars represent 400 μm. Error bars indicate mean ± SD. *p < 0.05; **p <0.01, ***p < 0.005.

Supplementary Figure 3

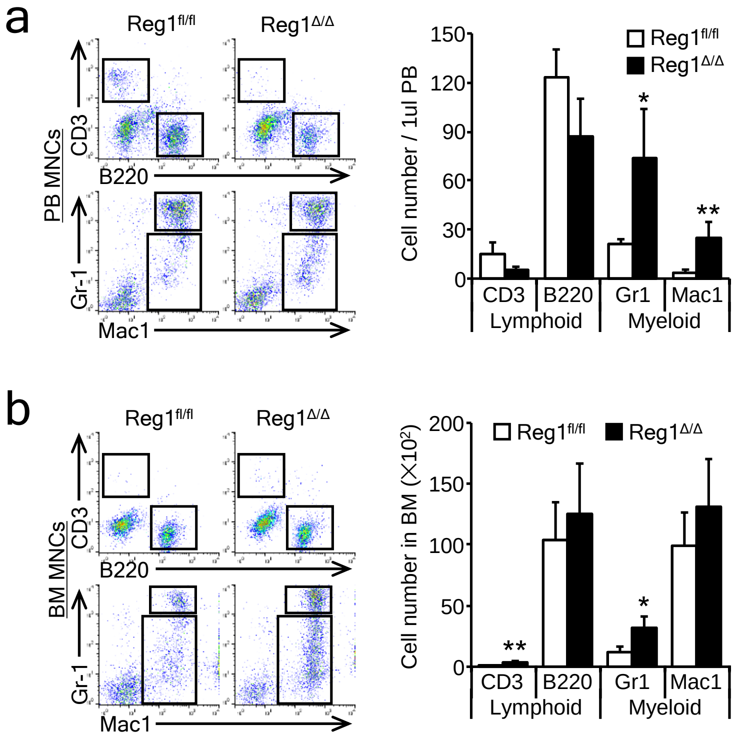

Supplementary Figure 3. Analysis of peripheral blood and BM cells of juvenile Regnase-1-deficient mice.

(**a**) Flow cytometric analysis of lymphocytes and monocytes in peripheral blood mononuclear cells (MNCs) from 2 week-old mice as indicated (n =3 per group; 2 independent experiments). Quantification of lymphoid and myeloid cells in peripheral blood MNCs. (**b**) Flow cytometric analysis of lymphocytes and monocytes in BM MNCs from 2 week-old mice as indicated (n =3 per group; 2 independent experiments). Quantification of lymphoid and myeloid cells in BM MNCs. Error bars indicate mean ± SD. *$p < 0.05$; **$p < 0.01$, t-test.

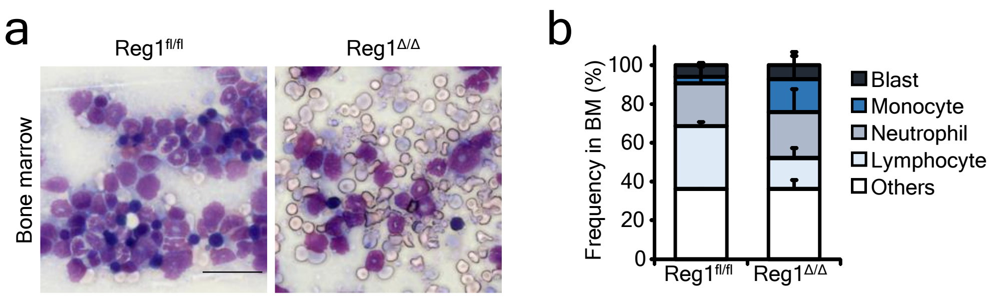

c

		frequency(%)	SD	p value
Blast	Reg1$^{fl/fl}$	0.0	0.6	0.001
	Reg1$^{\Delta/\Delta}$	2.6	1.8	

		normal			abnormal		
		frequency(%)	SD	p value	frequency(%)	SD	p value
Neutrophil	Reg1$^{fl/fl}$	22.0	7.1	0.001	0.0	0.0	0.003
	Reg1$^{\Delta/\Delta}$	45.0	11.3		3.2	2.0	
Monocyte	Reg1$^{fl/fl}$	4.3	1.5	0.449	0.6	0.8	<0.001
	Reg1$^{\Delta/\Delta}$	3.7	1.8		9.4	3.0	
Lymphocyte	Reg1$^{fl/fl}$	71.4	7.5	<0.001	0.4	0.8	0.008
	Reg1$^{\Delta/\Delta}$	30.5	11.8		4.2	2.7	

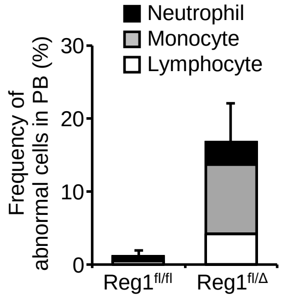

d

		frequency(%)	SD	p value
Blast	Reg1$^{fl/fl}$	5.9	1.9	0.299
	Reg1$^{\Delta/\Delta}$	7.2	1.3	

		normal			abnormal		
		frequency(%)	SD	p value	frequency(%)	SD	p value
Neutrophil	Reg1$^{fl/fl}$	22.1	6.8	0.769	0.0	0.0	0.073
	Reg1$^{\Delta/\Delta}$	20.8	5.6		3.0	2.7	
Monocyte	Reg1$^{fl/fl}$	3.4	0.9	0.001	0.0	0.0	0.146
	Reg1$^{\Delta/\Delta}$	14.9	2.7		2.1	2.5	
Lymphocyte	Reg1$^{fl/fl}$	32.2	8.1	0.002	0.3	0.3	0.007
	Reg1$^{\Delta/\Delta}$	9.1	3.7		6.9	3.3	

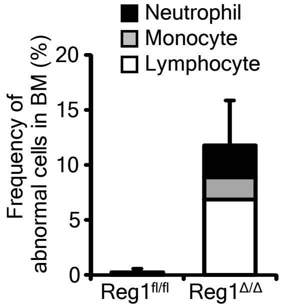

Supplementary Figure 4. Pathological analysis of BM cell of Regnase1-deficient mice.
(**a**) Representative field of Wright-Giemsa-stained BM smears of 8 week-old control (fl/fl) and Regnase-1-KO (Δ/Δ) mice. The scale bar shows 30 μm. (**b**) Quantification of frequencies of blasts and other MNCs BM on Wright-Giemsa-staining (n =3 per group; 3 independent experiments). (**c**) Quantification of abnormal cells in the PB of Reg1$^{fl/\Delta}$ mice (n = 6 per group). (**d**) Quantification of abnormal cells in the BM of Reg1$^{\Delta/\Delta}$ mice (n = 3 per group; 2 independent experiments).

Supplementary Figure 5

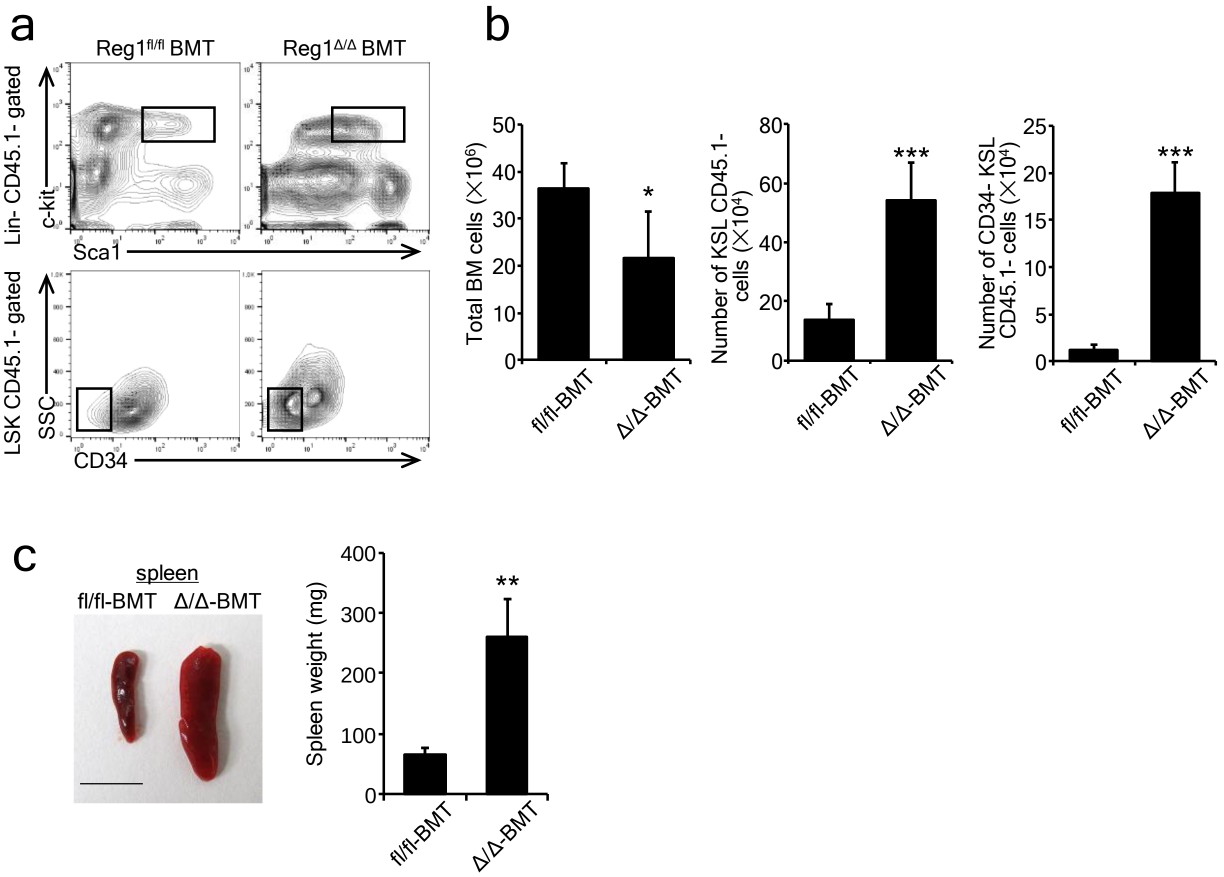

d

		frequency(%)	SD	p value
Blast	$Reg1^{fl/fl}$	0.0	0.0	-
	$Reg1^{\Delta/\Delta}$	0.0	0.0	

		normal			abnormal		
		frequency(%)	SD	p value	frequency(%)	SD	p value
Neutrophil	$Reg1^{fl/fl}$	22.0	8.7	0.242	0.3	0.6	0.000
	$Reg1^{\Delta/\Delta}$	29.0	1.7		19.2	1.8	
Monocyte	$Reg1^{fl/fl}$	1.2	1.2	0.624	1.2	1.2	0.049
	$Reg1^{\Delta/\Delta}$	0.7	1.3		4.0	1.3	
Lymphocyte	$Reg1^{fl/fl}$	71.8	8.1	0.001	3.5	1.3	0.001
	$Reg1^{\Delta/\Delta}$	24.0	1.3		23.0	3.9	

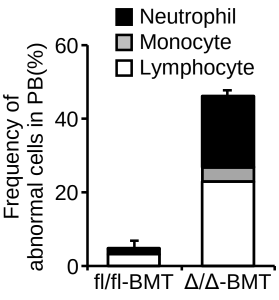

Supplementary Figure 5. Transplantation of leukemic BM mononuclear cells of Regnase-1-deficient mice to wild-type mice.
WT recipients (CD45.1) transplanted with 1×10^7 BM mononuclear cells of either control (fl/fl) or Regnase-1-KO (Δ/Δ) mice without irradiation. Representative flow cytometric analysis (**a**), quantitative and statistical analyses of BM cells and HSCs (**b**) in the BM of mice 12 weeks after transplantation (n = 4 per group; 2 independent experiments). (**c**) Representative gross appearance of the spleens from transplanted mice as indicated. The scale bars represent 1 cm. (**d**) Quantification of abnormal cells in the PB of mice 12 weeks after transplantation (n = 3 per group; 2 independent experiments). Error bars indicate mean ± SD. *$p < 0.05$; **$p < 0.01$, ***$p < 0.005$, t-test.

Supplementary Figure 6

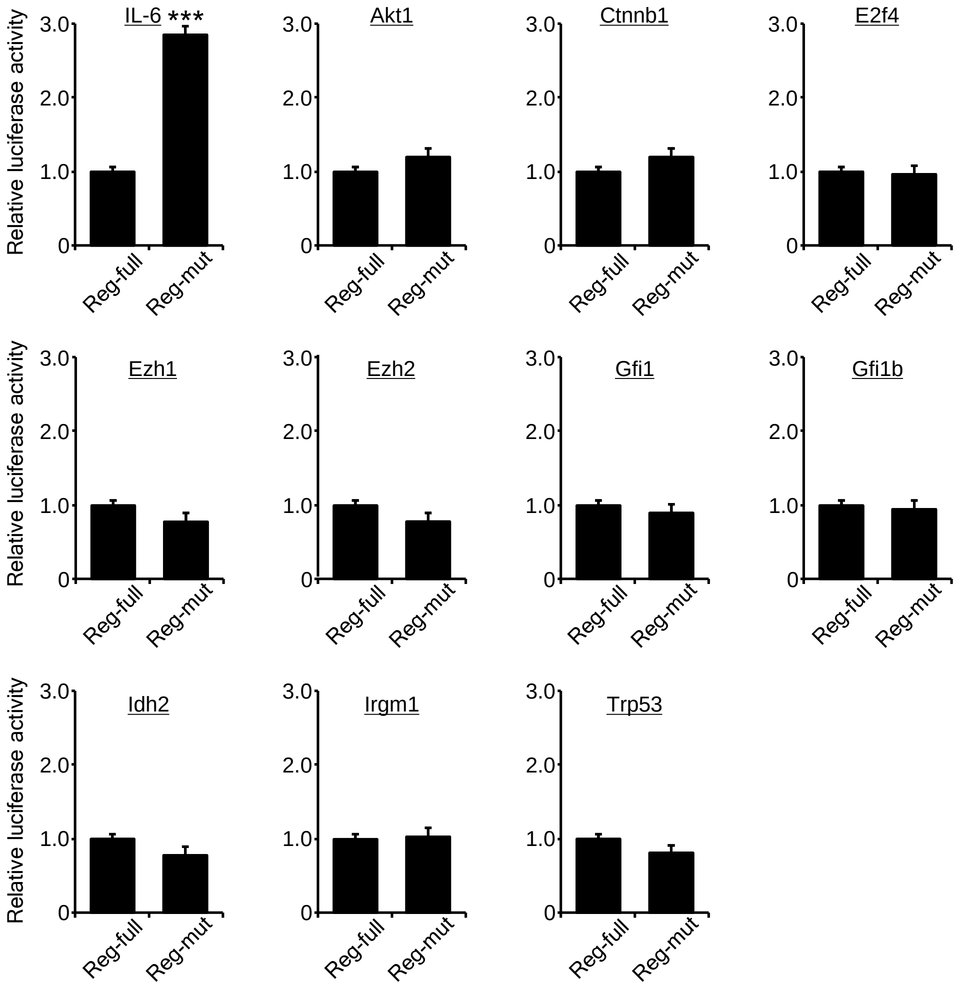

Supplementary Figure 6. Identification of Regnase-1 target mRNAs in CD34$^-$ HSCs. Luciferase activity of HEK293 cells transfected with luciferase reporter plasmids containing 3′UTRs of the indicated genes and either Regnase-1 expression plasmid or inactive Regnase-1 mutant plasmid (3 independent experiments). 3′UTR of *IL-6* was used as a positive control. Error bars indicate mean ± SD. ***p < 0.005, t-test.

Supplementary Figure 7

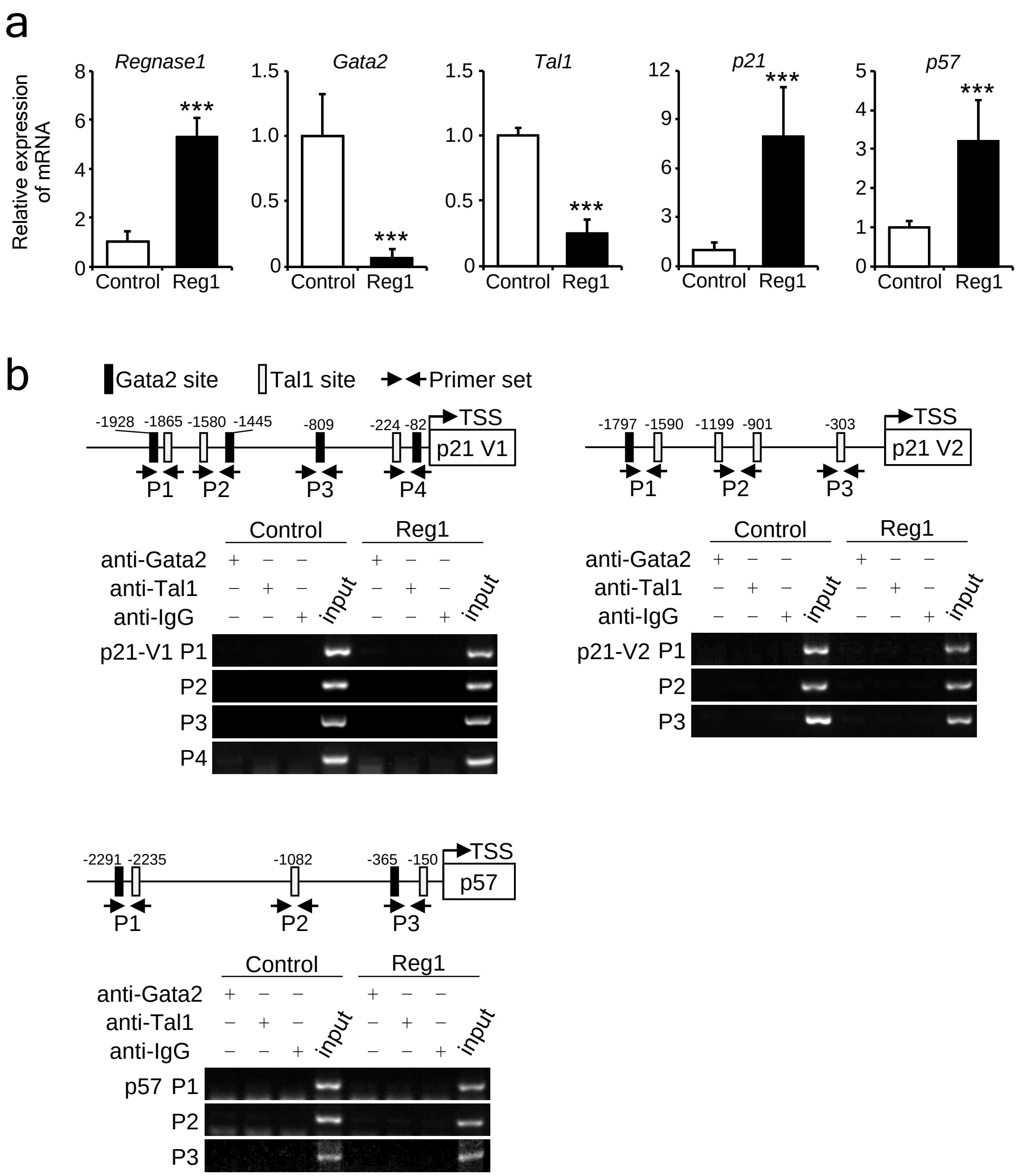

Supplementary Figure 7. Examination of direct associations between Gata2/Tal2 and p21/p57. (**a**) qRT-PCR analysis of *Regnase-1*, *Gata2*, *Tal1*, *p21* and *p57* expression in THP1 cells transfected with Regnase-1 expression vector (Reg1) or control mock vector. Data are expressed as fold-change relative to control vector transfection (n =3 per group). Error bars indicate mean ± SD. *** $p < 0.005$, t-test. (**b**) Schematic representation of the positions of primers, transcription factor binding sites, and part of the first intron in the indicated human genes. The black box represents Gata2-binding sites and the white box represents Tal1 binding sites. Positions of primers used for ChIP are designated by arrows. Chromatin extracts from Regnase1-overexpressing or control THP1 cells were immunoprecipitated with an anti-human Gata2 antibody, Tal1 antibody or negative control IgG antibody. Immunoprecipitated promoter sequences were analyzed by PCR. Input DNA served as the technical positive control for PCR amplification.

Supplementary Figure 8

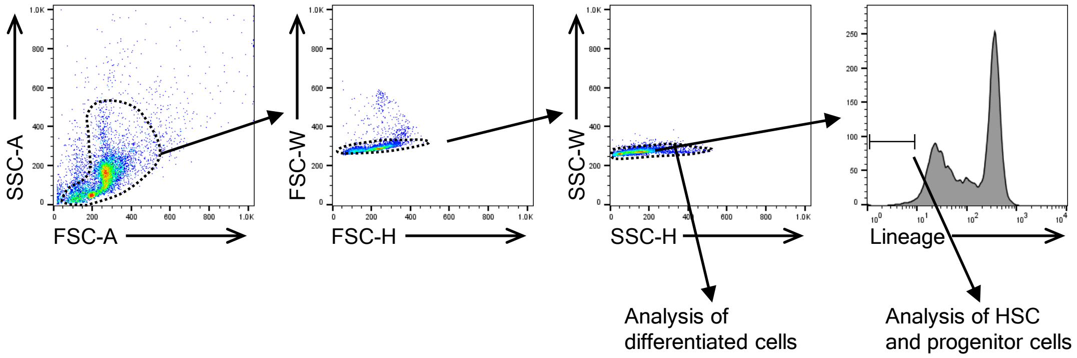

Supplementary Figure 8.Gating strategies used for cell analysis and sorting.
Gating strategy to analyze and BM cells from control (fl/fl) and Regnase-1-KO (Δ/Δ) mice (Fig.1c,e,f, Fig.2a-d, f-I, Fig.3a,b, e,f,h,I,k,l, Fig.4d-g,k-m and Fig.7c-f, h,i).
