# Supplementary material for: Regnase-1-mediated post-transcriptional regulation is essential for hematopoietic stem and progenitor cell homeostasis
Source: Nat Commun. 2019 Mar 6;10:1072. doi: 10.1038/s41467-019-09028-w (PMC6403248; doi:10.1038/s41467-019-09028-w)
Supplement: Supplementary file 3 — Description of Additional Supplementary Files [file 41467_2019_9028_MOESM3_ESM.pdf]

Description of Additional Supplementary Files

Supplementary Data 1
Association of abnormal expression of Regnase-1 (ZC3H12A) mRNA and prognosis of cancer patients

Supplementary Data 2
List of primer and Oligonucleotide sequences.

Supplementary Data 3
Result of RNA-seq analysis of $CD34^{-}$ HSCs from Vav1-iCre; $Reg1^{flox/flox}$ mice (KO) and control $Reg1^{flox/flox}$ mice (Control).

Supplementary Data 4
The gene sets identified by RNA-seq analysis used for the gene ontology analysis in Figure 2j.

Supplementary Data 5
RNA-seq data in Figure 5g,h
Pre-defined gene sets which are related to leukemia used for the gene set enrichment analysis in Figure 5g,h.

Supplementary Data 6
Summarized results of gene set enrichment analysis in Figure 5g,h.
