# Supplementary material for: Regnase-1-mediated post-transcriptional regulation is essential for hematopoietic stem and progenitor cell homeostasis
Source: Nat Commun. 2019 Mar 6;10:1072. doi: 10.1038/s41467-019-09028-w (PMC6403248; doi:10.1038/s41467-019-09028-w)
Supplement: Supplementary file 10 — Reporting Summary [file 41467_2019_9028_MOESM10_ESM.pdf]

nature research

Corresponding author(s): Nobuyuki Takakura

Last updated by author(s): Jan 28, 2019

Reporting Summary

Nature Research wishes to improve the reproducibility of the work that we publish. This form provides structure for consistency and transparency in reporting. For further information on Nature Research policies, see Authors & Referees and the Editorial Policy Checklist.

Statistics

For all statistical analyses, confirm that the following items are present in the figure legend, table legend, main text, or Methods section.

n/a	Confirmed	
☐	☒	The exact sample size (*n*) for each experimental group/condition, given as a discrete number and unit of measurement
☐	☒	A statement on whether measurements were taken from distinct samples or whether the same sample was measured repeatedly
☐	☒	The statistical test(s) used AND whether they are one- or two-sided<br>*Only common tests should be described solely by name; describe more complex techniques in the Methods section.*
☐	☒	A description of all covariates tested
☐	☒	A description of any assumptions or corrections, such as tests of normality and adjustment for multiple comparisons
☐	☒	A full description of the statistical parameters including central tendency (e.g. means) or other basic estimates (e.g. regression coefficient) AND variation (e.g. standard deviation) or associated estimates of uncertainty (e.g. confidence intervals)
☐	☒	For null hypothesis testing, the test statistic (e.g. *F*, *t*, *r*) with confidence intervals, effect sizes, degrees of freedom and *P* value noted<br>*Give P values as exact values whenever suitable.*
☒	☐	For Bayesian analysis, information on the choice of priors and Markov chain Monte Carlo settings
☒	☐	For hierarchical and complex designs, identification of the appropriate level for tests and full reporting of outcomes
☒	☐	Estimates of effect sizes (e.g. Cohen's *d*, Pearson's *r*), indicating how they were calculated

Our web collection on statistics for biologists contains articles on many of the points above.

Software and code

Policy information about availability of computer code

Data collection	*Provide a description of all commercial, open source and custom code used to collect the data in this study, specifying the version used OR state that no software was used.*
Data analysis	*Provide a description of all commercial, open source and custom code used to analyse the data in this study, specifying the version used OR state that no software was used.*

For manuscripts utilizing custom algorithms or software that are central to the research but not yet described in published literature, software must be made available to editors/reviewers. We strongly encourage code deposition in a community repository (e.g. GitHub). See the Nature Research guidelines for submitting code & software for further information.

Data

Policy information about availability of data

All manuscripts must include a data availability statement. This statement should provide the following information, where applicable:

- Accession codes, unique identifiers, or web links for publicly available datasets
- A list of figures that have associated raw data
- A description of any restrictions on data availability

We have provided a full data availability statement in the manuscript.

Field-specific reporting

Please select the one below that is the best fit for your research. If you are not sure, read the appropriate sections before making your selection.

☐ Life sciences ☐ Behavioural & social sciences ☐ Ecological, evolutionary & environmental sciences

Life sciences study design

All studies must disclose on these points even when the disclosure is negative.

Sample size	The number of independent samples analyses is provided for each experiment in the figure legend (between n=3 and n=6 independent samples for each group). Below is a summary of the sample size considerations for each figure.<br>For histological analysis (Figure 1d, j, 3c), at least femur samples collected from at least 3 independent wild-type (and mutant) litters were processed for immunohistochemical staining and embedded in one block for each sample.<br>For flow cytometric analysis (Figure 1f-i, 2a-d, 2f-i, 3a-b, 3e-f, 3k-l, 4d-g, 4k-l, 7c-f, 7h), sample size was determined based on the number of mutant mouse available in same age and similar weight.
Data exclusions	None of the data were excluded from the analyses. Reported in Methods 'Statistical analysis' subsection.
Replication	We have used at least 3 biological replicates for each experiment. This is designed to account for biological variability taking into account that the majority of experiments were performed in similar experiments.
Randomization	Mice of the same age and similar weight were randomly assigned to experimental or control groups
Blinding	Investigators were not blinded to allocation during cell and mouse assays. Reported in Methods 'Statistical analysis' subsection. It was not possible to be blinded the mice due to the obvious morphological differences between wild-type and mutant.

Behavioural & social sciences study design

All studies must disclose on these points even when the disclosure is negative.

Study description	*Briefly describe the study type including whether data are quantitative, qualitative, or mixed-methods (e.g. qualitative cross-sectional, quantitative experimental, mixed-methods case study).*
Research sample	*State the research sample (e.g. Harvard university undergraduates, villagers in rural India) and provide relevant demographic information (e.g. age, sex) and indicate whether the sample is representative. Provide a rationale for the study sample chosen. For studies involving existing datasets, please describe the dataset and source.*
Sampling strategy	*Describe the sampling procedure (e.g. random, snowball, stratified, convenience). Describe the statistical methods that were used to predetermine sample size OR if no sample-size calculation was performed, describe how sample sizes were chosen and provide a rationale for why these sample sizes are sufficient. For qualitative data, please indicate whether data saturation was considered, and what criteria were used to decide that no further sampling was needed.*
Data collection	*Provide details about the data collection procedure, including the instruments or devices used to record the data (e.g. pen and paper, computer, eye tracker, video or audio equipment) whether anyone was present besides the participant(s) and the researcher, and whether the researcher was blind to experimental condition and/or the study hypothesis during data collection.*
Timing	*Indicate the start and stop dates of data collection. If there is a gap between collection periods, state the dates for each sample cohort.*
Data exclusions	*If no data were excluded from the analyses, state so OR if data were excluded, provide the exact number of exclusions and the rationale behind them, indicating whether exclusion criteria were pre-established.*
Non-participation	*State how many participants dropped out/declined participation and the reason(s) given OR provide response rate OR state that no participants dropped out/declined participation.*
Randomization	*If participants were not allocated into experimental groups, state so OR describe how participants were allocated to groups, and if allocation was not random, describe how covariates were controlled.*

Ecological, evolutionary & environmental sciences study design

All studies must disclose on these points even when the disclosure is negative.

Study description	*Briefly describe the study. For quantitative data include treatment factors and interactions, design structure (e.g. factorial, nested, hierarchical), nature and number of experimental units and replicates.*
Research sample	*Describe the research sample (e.g. a group of tagged Passer domesticus, all Stenocereus thurberi within Organ Pipe Cactus National Monument), and provide a rationale for the sample choice. When relevant, describe the organism taxa, source, sex, age range and any manipulations. State what population the sample is meant to represent when applicable. For studies involving existing datasets, describe the data and its source.*

Sampling strategy | *Note the sampling procedure. Describe the statistical methods that were used to predetermine sample size OR if no sample-size calculation was performed, describe how sample sizes were chosen and provide a rationale for why these sample sizes are sufficient.*

Data collection | *Describe the data collection procedure, including who recorded the data and how.*

Timing and spatial scale | *Indicate the start and stop dates of data collection, noting the frequency and periodicity of sampling and providing a rationale for these choices. If there is a gap between collection periods, state the dates for each sample cohort. Specify the spatial scale from which the data are taken*

Data exclusions | *If no data were excluded from the analyses, state so OR if data were excluded, describe the exclusions and the rationale behind them, indicating whether exclusion criteria were pre-established.*

Reproducibility | *Describe the measures taken to verify the reproducibility of experimental findings. For each experiment, note whether any attempts to repeat the experiment failed OR state that all attempts to repeat the experiment were successful.*

Randomization | *Describe how samples/organisms/participants were allocated into groups. If allocation was not random, describe how covariates were controlled. If this is not relevant to your study, explain why.*

Blinding | *Describe the extent of blinding used during data acquisition and analysis. If blinding was not possible, describe why OR explain why blinding was not relevant to your study.*

Did the study involve field work? ☐ Yes ☐ No

Field work, collection and transport

Field conditions | *Describe the study conditions for field work, providing relevant parameters (e.g. temperature, rainfall).*

Location | *State the location of the sampling or experiment, providing relevant parameters (e.g. latitude and longitude, elevation, water depth).*

Access and import/export | *Describe the efforts you have made to access habitats and to collect and import/export your samples in a responsible manner and in compliance with local, national and international laws, noting any permits that were obtained (give the name of the issuing authority, the date of issue, and any identifying information).*

Disturbance | *Describe any disturbance caused by the study and how it was minimized.*

Reporting for specific materials, systems and methods

We require information from authors about some types of materials, experimental systems and methods used in many studies. Here, indicate whether each material, system or method listed is relevant to your study. If you are not sure if a list item applies to your research, read the appropriate section before selecting a response.

Materials & experimental systems

n/a	Involved in the study	
☐	☒	Antibodies
☐	☒	Eukaryotic cell lines
☒	☐	Palaeontology
☐	☒	Animals and other organisms
☒	☐	Human research participants
☒	☐	Clinical data

Methods

n/a	Involved in the study	
☒	☐	ChIP-seq
☐	☒	Flow cytometry
☒	☐	MRI-based neuroimaging

Antibodies

Antibodies used | Anti-cKit (AF1356, R&D Systems), anti-Sca1 (553333, BD Biosciences), anti-CD71 (113802, Biolegend), anti-Gata2 (ab22849, Abcam) anti-Tal1(C15200012, Diagenode), anti-Regnase-1, fluorescence-labeled anti-CD34 (12-0349-42, eBioscience), -Flk2/CD135 (135309, BioLegend), -Sca-1 (108127, BioLegend), -c-Kit (105825, BioLegend), -FcγR (101325, BioLegend), -IL7Rα (121111,BioLegend), -CD71 (113807, BioLegend), -CD3 (100205, BioLegend), -Mac1 (101205, BioLegend), -B220 (103205, BioLegend), -Gr1 (108407, BioLegend), -CD45 (103134, BioLegend), -CD93 (136503, BioLegend), -CD150 (115909, BioLegend), -CD48 (103403, BioLegend), -CD45.1(110730, BioLegend) and -lineage Cocktail antibodies (133301, BioLegend).

Validation | All antibodies except anti-Regnase-1 antibody were validated by manufacturer.Anti-Regnase-1 antibody was validated in previous report (Iwasaki H. et al. Nat Immunol. 2011. Uehata, T. et al. Cell. 2013).

Eukaryotic cell lines

Policy information about cell lines

Cell line source(s)	All cell lines were provided by the RIKEN cell bank.
Authentication	All cell lines were authenticated by RIKEN cell bank.
Mycoplasma contamination	They were tested and proved negative. Reported in Methods 'Cell culture and transfections' subsection.
Commonly misidentified lines (See ICLAC register)	None.

Animals and other organisms

Policy information about studies involving animals; ARRIVE guidelines recommended for reporting animal research

Laboratory animals	C57BL/6 mice (B6-Ly5.2) were purchased from Japan SLC (Shizuoka, Japan) and C57BL/6-Ly5.1 mice were purchased from Sankyo Labo Service (Tsukuba, Japan) and used between 6 and 12 weeks of age.
Wild animals	*Provide details on animals observed in or captured in the field; report species, sex and age where possible. Describe how animals were caught and transported and what happened to captive animals after the study (if killed, explain why and describe method; if released, say where and when) OR state that the study did not involve wild animals.*
Field-collected samples	*For laboratory work with field-collected samples, describe all relevant parameters such as housing, maintenance, temperature, photoperiod and end-of-experiment protocol OR state that the study did not involve samples collected from the field.*
Ethics oversight	All experiments were carried out under the guidelines of Osaka University Committee for animal and recombinant DNA experiments and were approved by the Osaka University Institutional Review Board.

Note that full information on the approval of the study protocol must also be provided in the manuscript.

Flow Cytometry

Plots

Confirm that:

☒ The axis labels state the marker and fluorochrome used (e.g. CD4-FITC).

☒ The axis scales are clearly visible. Include numbers along axes only for bottom left plot of group (a 'group' is an analysis of identical markers).

☒ All plots are contour plots with outliers or pseudocolor plots.

☒ A numerical value for number of cells or percentage (with statistics) is provided.

Methodology

Sample preparation	Fetal liver and bone marrow were dissected form mice and cells harvested by pipetting. Debris and aggregated cells were removed through a nylon-mesh.
Instrument	FACS Aria (BD Biosciences)
Software	FlowJo software (TreeStar)
Cell population abundance	Abundance of relevant cell population within post-sort fraction and purity of sorted cell population was validated from re-analysis by flow cytometry.
Gating strategy	Gating strategy was indicated in Supplementary Figure8 and each Figures.

☒ Tick this box to confirm that a figure exemplifying the gating strategy is provided in the Supplementary Information.
